# Quack Doctors and Medicines

**Published:** 1847-02

**Authors:** 


*Quack Doctors and Medicines.*—The Mayor of Lyons has just issued a proclamation that no bills or placards announcing the treatment of any disease by particular individuals, or the sale of any particular medicines, shall be posted on any of the walls of the city, or otherwise exposed to public view; and further that none of the public newspapers shall insert any such announcement in their advertisements or otherwise. A similar ordinance has been issued in Paris and every city in France.—*Jour. de Chem. Med.*

